# Characterization of pH dependent Mn(II) oxidation strategies and formation of a bixbyite-like phase by *Mesorhizobium australicum* T-G1

**DOI:** 10.3389/fmicb.2015.00734

**Published:** 2015-07-17

**Authors:** Tsing Bohu, Cara M. Santelli, Denise M. Akob, Thomas R. Neu, Valerian Ciobota, Petra Rösch, Jürgen Popp, Sándor Nietzsche, Kirsten Küsel

**Affiliations:** ^1^Department of Aquatic Geomicrobiology, Friedrich Schiller University JenaJena, Germany; ^2^Department of Mineral Sciences, Smithsonian InstitutionWashington, DC, USA; ^3^National Research Program, United States Geological SurveyReston, VA, USA; ^4^Department of River Ecology, Helmholtz Centre for Environmental Research-UFZMagdeburg, Germany; ^5^Institute of Physical Chemistry and Abbe School of Photonics, Friedrich Schiller University JenaJena, Germany; ^6^Leibniz Institute of Photonic TechnologiesJena, Germany; ^7^Centre of Electron Microscopy, University Hospital Jena, Friedrich Schiller University JenaJena, Germany; ^8^German Centre for Integrative Biodiversity Research (iDiv) Halle-Jena-LeipzigLeipzig, Germany

**Keywords:** catalase, low pH, Mn(II) oxidation, multi-copper oxidase, reactive oxygen species

## Abstract

Despite the ubiquity of Mn oxides in natural environments, there are only a few observations of biological Mn(II) oxidation at pH < 6. The lack of low pH Mn-oxidizing bacteria (MOB) isolates limits our understanding of how pH influences biological Mn(II) oxidation in extreme environments. Here, we report that a novel MOB isolate, *Mesorhizobium australicum* strain T-G1, isolated from an acidic and metalliferous uranium mining area, can oxidize Mn(II) at both acidic and neutral pH using different enzymatic pathways. X-ray diffraction, Raman spectroscopy, and scanning electron microscopy with energy dispersive X-ray spectroscopy revealed that T-G1 initiated bixbyite-like Mn oxide formation at pH 5.5 which coincided with multi-copper oxidase expression from early exponential phase to late stationary phase. In contrast, reactive oxygen species (ROS), particularly superoxide, appeared to be more important for T-G1 mediated Mn(II) oxidation at neutral pH. ROS was produced in parallel with the occurrence of Mn(II) oxidation at pH 7.2 from early stationary phase. Solid phase Mn oxides did not precipitate, which is consistent with the presence of a high amount of H_2_O_2_ and lower activity of catalase in the liquid culture at pH 7.2. Our results show that *M. australicum* T-G1, an acid tolerant MOB, can initiate Mn(II) oxidation by varying its oxidation mechanisms depending on the pH and may play an important role in low pH manganese biogeochemical cycling.

## Introduction

A variety of Mn(III/IV) oxide/hydroxide minerals are ubiquitous on Earth (Tebo et al., 2004). In terrestrial environments, birnessite, lithiophorite, and hollandite are the most common Mn oxides (Lind and Hem, 1993; Post, 1999). Mn oxides are thought to be primarily of biological origin because abiotic Mn(II) oxidation is slow in most natural environments (Nealson et al., 1988). Bacteria and fungi are known to catalyze Mn oxidation, with the majority of Mn oxidizers cultivated under neutral to slightly alkaline pH (Francis and Tebo, 1999; Francis et al., 2001; Anderson et al., 2009; Santelli et al., 2010). However, in acidic environments, Mn oxides are present (Burkhardt et al., 2009; Grawunder et al., 2009) although their abiotic formation is not thermodynamically favorable (Tebo et al., 2004; Nealson, 2006; Luther, 2010). Recently, Mn(II)-oxidizing bacteria (MOB) were isolated at low pH (Akob et al., 2014), however, the mechanism of biological Mn(II) oxidation at acidic pH is unknown.

Low pH may negatively affect both abiotic and biotic Mn oxidation. Unlike neutral or alkaline conditions, oxidation in acidic environments is predicted to be thermodynamically unfavorable for initiating Mn oxide formation when O_2_ is the terminal electron acceptor (Sato, 1960). Additional energy would be needed to support the membrane potential at acidic pH (Mitchell, 1961), thus limiting the rate of biological Mn(II) oxidation from which microbes are not known to gain energy (Bargar et al., 2005). The availability of negatively charged sites is also greatly reduced under acidic conditions. Fewer metal ions are consequently immobilized which may inhibit Mn(II) adsorption by competition (Gadde and Laitinen, 1974). Furthermore, low pH may inhibit the expression of genes involved in biological Mn(II) oxidation, as seen for other genes under low pH conditions (Beales, 2004).

Heterotrophic organisms catalyze Mn oxidation, and both direct and indirect mechanisms are identified for Mn oxidation by model MOB isolated at circumneutral pH (Tebo et al., 2004). Direct enzymatic oxidation is mainly linked to multi-copper oxidases (MCO; Francis and Tebo, 2002), whereas reactive oxygen species (ROS) are involved in indirect enzymatic Mn(II) oxidation (Learman et al., 2011; Hansel et al., 2012). MCOs are a family of enzymes with various structures to oxidize a number of substrates including lignin (Larrondo et al., 2003) and humic acids (Morin et al., 2012). The MCOs catalyze the two steps involved in the one electron transfer from Mn(II) to Mn(III/IV) (Dick et al., 2008; Soldatova et al., 2012). Additionally, Mn(III) can also disproportionate immediately to Mn(II) and Mn(IV) or exist complexed with ligands in natural environments (Tebo et al., 2005). ROS are unavoidable by-products of aerobic metabolism (Aguirre et al., 2005) and encompass molecules such as hydrogen peroxide, radicals (e.g., the hydroxyl radical), and the superoxide anion. Production of superoxides can be induced by a variety of biotic or abiotic stresses (Cakmak, 2000; Diaz et al., 2013). Bacteria were shown to indirectly oxidize Mn(II) through the enzymatic generation of superoxides (Learman et al., 2011). However, it is not known if these mechanisms are used in bacterial Mn(II) oxidation at acidic pH.

The ecological and practical importance of low pH Mn oxidation may be more significant than often acknowledged. In addition to contributing to biogeochemical cycling of carbon and Mn, low pH Mn oxidation may be especially important for bioremediation of heavy metals in acidic environments, such as that seen in an acidic and metalliferous uranium mining area where Mn-rich geochemical barriers are involved in heavy metal natural attenuation (Burkhardt et al., 2009; Akob et al., 2014). As microorganisms are responsible for forming the majority of Mn oxide minerals in natural environments and little is known about low pH microbial Mn(II) oxidation, we isolated a novel MOB, *Mesorhizobium australicum* T-G1, from the acidic and heavy metal-contaminated inactive Ronneburg uranium mining area. We linked this organism to biogenic bixbyite-like Mn oxide formation under acidic conditions and elucidated that T-G1 could utilize a binary strategy to oxidize Mn(II) depending on the acidic or neutral pH conditions.

## Materials and Methods

### Site and Sampling Description

The sampling site was located at the former uranium leaching heap (location E 4510469, N 5635476, Gauss-Krueger Potsdam coordinate system) within the Ronneburg mining district. It was one of the most important uranium mining areas in the former German Democratic Republic. Uranium mining at this district started in 1951 and ceased in 1990. Black shale containing low concentration of uranium was leached with acid mine drainage (AMD) and sulfuric acid from the 1970s to 1989. Site remediation, including relocation of the heap material and capping the area with allochthonous soil, was completed in 2004 although contaminated groundwater still threatens nearby ecosystems; for a more detailed description of the site history see (Grawunder et al., 2009; Schäffner, 2013).

A subsurface soil layer enriched in Mn oxide and hydroxide minerals is present at the former leaching heap (Burkhardt et al., 2009; Akob et al., 2014). The Mn oxides were identified as birnessite and todorokite by XRD, microprobe analyses and transmission electron microscopy (TEM) with pre-separation against the density of the minerals (Schäffner, 2013).

Sediment samples were collected aseptically from this Mn-rich layer at a depth of 70 cm below the surface in November 2011 using a soil corer (PKH-100, MMM tech support, Germany). Samples were stored on ice for transport and inoculated into culture media on the same day. The pH of the sediment was measured using the CaCl_2_ method as previously described (Schofield and Taylor, 1955). Other physicochemical parameters of this Mn-rich layer including pH of the pore water can be retrieved from (Burkhardt et al., 2009) and also briefly summarized in Supplementary Table S1.

### Isolation and Identification of Strain T-G1

Strain T-G1 was isolated from a 10^-1^ dilution in 0.7% NaCl of the Mn-rich layer sample on modified low pH PYG agar plates. PYG agar (Emerson and Ghiorse, 1992) contained per liter, 0.25 g peptone, 0.25 g yeast extract, 0.25 g glucose, 20 g agar, and 200 μM MnCl_2_, and were buffered to pH 5.5 with 10 mM 2-(*N*-morpholino) ethanesulfonic acid (MES, Sigma, USA). Plates were incubated at 15°C in the dark. Single colonies were transferred five times by plate streaking. The leucoberbelin blue (LBB) spot test was used to identify Mn(II) oxides as described previously (Krumbein and Altmann, 1973). Briefly, 0.04% LBB was dropped on the colony surface and the presence of a blue color change on or near the colony was detected visually. Cell morphology was observed by epifluorescence microscopy (Axioplan, Zeiss, Germany) with the nucleic acid stain Syto 13 (Molecular Probes, Eugene, OR, USA) and field emission scanning electron microscopy (SEM; LEO-1530 Gemini, Carl Zeiss NTS GmbH, Germany, electron energy = 10 keV, secondary electron detector). The pH of the liquid culture was monitored during the incubation with a pH electrode (SenTix Mic, WTW). In addition, microscale pH changes were monitored in and around T-G1 colonies on agar plates using a pH microelectrode (Unisense, Denmark).

Genomic DNA was extracted from strain T-G1 using a sodium dodecyl sulfate (SDS)-based soil DNA extraction method slightly modified from (Zhou et al., 1996). Briefly, the DNA was extracted from liquid grown cultures using a bead-beating technique (4.0 ms^-1^ for 20 s, FastPrep-24^TM^ bead beater, MP Biomedicals, Canada) for cell lysis with chloroform/isoamyl alcohol purification and isopropanol precipitation.

The DNA extract (1 μl) was polymerase chain reaction (PCR) amplified using universal bacterial 16S rRNA gene primers 27F and 1492R, as described previously (Lane, 1991). Amplicons were purified using the GeneJET Gel Extraction and DNA Cleanup Micro kit (Thermo Scientific, Lithuania), according to the manufacturers’ instructions. Bidirectional Sanger sequencing was performed at Macrogen Inc. (Seoul, Republic of Korea) with primers 27F and 1492R. Sequences were assembled using Geneious Pro version 4.6.0 (Drummond et al., 2009). Sequence similarity was determined using the BLAST algorithm against the GenBank database available from NCBI (Altschul et al., 1990). Phylogenetic trees were constructed with the neighbor-joining algorithm using the MEGA 5.2 software package (Tamura et al., 2011).

### Biogenic Mineral Characterization

The biogenic minerals formed by strain T-G1 were harvested from liquid cultures grown in PYG media supplemented with varying MnSO_4_ concentrations (0.1, 1, or 10 mM) and buffered to pH 5.5 with 10 mM MES (Sigma, USA). The liquid cultures were incubated at 15°C in the dark without shaking. The biogenic minerals were characterized using scanning electron microscopy with energy dispersive X-ray spectroscopy (SEM-EDS), Raman spectroscopy and XRD. For SEM, 1 ml of the above liquid culture was centrifuged at 3000 × *g* for 5 min to collect the cell-mineral pellet. The pellet was then fixed with 2.5% glutaraldehyde in 1 × PBS at pH 7.4, followed by washing three times with 1 × PBS (Carmichael et al., 2013). A subsample of the biogenic Mn mineral was directly aliquoted onto adhesive conductive carbon tabs (Electron Microscopy Sciences, USA) and dehydrated for 5 min each in an ascending ethanol dehydration series (10, 20, 30, 50, 70, 80, 90, and 100%), then air dried overnight. Samples were examined uncoated at low vacuum (0.8 mp chamber pressure) in an Ultra-high resolution FEG-SEM (FEI Nova NanoSEM 600, USA). Elemental distribution was obtained using EDS (ThermoFisher, USA) fitted on the FEG-SEM.

Particles from the liquid cultures were investigated directly with Raman spectroscopy on a LabRam micro-spectrometer (Jobin Yvon Horiba) using a 532 nm monochromatic radiation from a frequency-doubled Nd:YAG laser (Reiche et al., 2011) as an excitation source. The laser beam of about 400 μW was focused on the samples by a Leica PLFluoar objective (100 × NA 0.75). The spectral resolution of the Raman spectrometer was around 6 cm^-1^. Spectra were recorded with the LabRaman system running the LabSpec software 3.01 and plotted in Origin 7.5. The Raman spectra were checked manually for the positions and relative intensities of the peaks and compared with published data. For XRD, 10 ml of the liquid culture described above was centrifuged at 3000 ×*g* for 5 min. The precipitate was air-dried, placed onto the tip of a thin glass fiber, and analyzed on a D/MAX RAPID microdiffractometer with an imagine plate detector (Rigaku, USA). Analysis was conducted with MoKα radiation at 40 mA and 50 kV, with a 0.3 mm collimator, and rotated 1° per second on the phi axis for a total count time of 10 min. Patterns were analyzed using the JADE 9 (Materials Data, Inc., USA) software and matched with known patterns from the International Center for Diffraction Data (ICDD) PDF-2 database (http://www.icdd.com/).

The interactions between hydrated isolate T-G1 cells and biogenic Mn mineral particles were observed in a hydrated sample using confocal laser scanning microscopy (CLSM). Cells were grown as described above for mineral characterization with no preservation prior to analysis. Bacterial cells were stained using the nucleic acid stain Syto 9 and the membrane stain FM 1-43 (Molecular Probes, Eugene, OR, USA). The TCS SP5X (Leica, Germany) was equipped with an upright microscope and a supercontinuum light source, controlled with the software LAS AF version 2.6.1. For excitation, the laser lines at 488 nm (Syto 9) and 479 nm (FM 1-43) were selected. Emission signals were collected, 483–493 nm and 474–483 nm (reflection), 500–560 nm (Syto 9) and 480-650 nm (FM 1-43). The dataset was decovolved using Huygens ver. 14.10 (SVI, The Netherlands). The image series presented was projected using Imaris ver.8.0 (Bitplane).

### Rates of Growth and Mn(II) Oxidation at Acidic and Neutral pH

Isolate T-G1 was grown to exponential phase (OD_600__nm_ = 0.2) in PYG liquid medium at pH 6.8. In 96-microwell suspension culture plates (polystyrene, flat bottom, Corning, NY, USA), 2 μl log phase T-G1 liquid culture was transferred to 300 μl fresh PYG medium adjusted to either pH 5.5 (buffered by 10 mM MES, Sigma) or 7.2 (buffered by 10 mM 4-(2-Hydroxyethyl)piperazine-1-ethanesulfonic acid, Sigma) with or without 100 μM MnSO_4_. Prior to growth measurements, ascorbic acid (200 μM) was applied to dissolve Mn(III/IV) oxides (Dick et al., 2006). Growth curves were obtained using a Synergy HT Multi-Mode Microplate Reader (BioTek Instruments, Inc., USA) with 13 measurements at 600 nm taken over the course of growth.

Mn(II) oxidation was monitored in different growth phases using a slightly modified LBB colorimetric method as described before (Lee and Tebo, 1994). Briefly, at each time point, 20 μl incubated culture was transferred to a 96-microwell plate and 60 μl 0.04% LBB was added prior to measurement on the plate reader at 618 nm. A standard curve to determine Mn (III/IV) concentration was prepared by the reaction of known concentrations of KMnO_4_ and LBB with the reagent volume ratio of 1:3. The rates were calculated as,

$$Linear\,\;rate=([Mn(III/IV){]}_{164.5}-[M(III/IV){]}_{24.5})/F$$

[Mn(III/IV)]_24.5_ was the measured Mn(III/IV) concentration at 24.5 h; [Mn(III/IV)]_164.5_ was the measured Mn(III/IV) concentration at 164.5 h; and *F* was the time over the linear increasing phase of Mn(III/IV). Here, *F* = 140 h. Each treatment had three biological replicates.

### Mn(III) Trapping Experiment

Mn(III)-pyrophosphate trapping experiment were conducted as described before (Learman et al., 2011). In brief, cell free filtrates of T-G1 were obtained by filtering exponential phase liquid cultures with 0.45 μM PVDF syringe filters (Rotilabo^®^, Carl Roth, Germany). Absorbance (480 nm) of the filtrate was monitored for 4 h after the addition of 100 μM sodium pyrophosphate using a Hach DR 3800 UV-VIS bench top spectrophotometer (Hach Lange GmbH, Germany). The reaction was stopped when the absorbance at 600 nm of the experimental sample (containing pyrophosphate) was greater than the control (no pyrophosphate). Standard curves were prepared with Mn(III) acetate and 100 μM pyrophosphate. Superoxide dismutase (SOD, Sigma, USA) and Proteinase K (Sigma, USA) were used as superoxides and enzyme inhibitors. The rates were calculated as,

$$Linear\;rate=([Mn(III){]}_{f}-[M(III){]}_{o})/F$$

[Mn(III)]_0_ was the measured Mn(III) concentration at *T*_0_; [Mn(III)]_f_ was the measured Mn(III) concentration at *T*_f_; and *F* was the time over the linear increasing phase of Mn(III). Here, *F* = 52 min. Each treatment had three biological replicates.

### In-Gel Assay for Superoxide and Enzyme Activity

*Mesorhizobium australicum* T-G1 was cultured in liquid PYG media without Mn(II) at pH 5.5 and pH 7.2 as described above. Bacterial cells were harvested during both the exponential (94.5 h) and stationary phases (164.5) for the gel assay and the following tests by centrifuging at 6000 ×*g* for 5 min. After washing in 1 × PBS buffer, cells were lysed by bead beating with 1 g silicon beads (Lysing Matrix A, MP Biomedicals, Canada) on a FastPrep-24^TM^ bead beater (MP Biomedicals, Canada) at 4.0 ms^-1^ for 20 s. Whole cell protein was precipitated and concentrated by the dialysis method (Prelog, 1964). Polyacrylamide gel electrophoresis (PAGE) and sodium dodecyl sulfate polyacrylamide gel electrophoresis (SDS-PAGE) were performed with Mini-PROTEAN^®^ Tetra Cell (Bio-Rad) at 200 V. After electrophoresis, the SDS-PAGE gel was stained separately with Coomassie Brilliant Blue R-250 staining solution (Bio-Rad, pH 1) and 200 μM MnSO_4_ (pH 6.81). Mn(II) oxidation was visualized by the formation of a brown Mn oxide band in the gel after 6 h of incubation (Francis and Tebo, 2002). For superoxide assays, the PAGE gel was incubated in a solution of diethylenetriaminepentaacetic dianhydride (DTPA; 40 μM) and nitroblue tetrazolium (NBT; 40 μM) overnight to detect superoxides. Superoxide was visualized by the formation of purple bands in the gel (Diaz et al., 2013).

### Superoxide Quantification

Superoxide was quantified with the chemiluminescent probe MCLA (2-methyl-6-2 (4-methoxyphenyl)-3,7-dihydroimidazo[1,2-a]pyrazin-3-one, Sigma, USA) in a 96-microwell plate assay following the protocol defined by Godrant et al. (2009). Cell free filtrates were prepared from cultures grown in exponential and stationary phase at pH 5.5 and 7.2. Luminescence data was collected on a Synergy HT Multi-Mode Microplate Reader (BioTek Instruments, Inc., USA). A reading was taken before the addition of MCLA to ensure that background luminescence was negligible. Then 12.5 μM of MCLA was added to all wells and luminescence was monitored; a steady value was observed after 30 s (Diaz et al., 2013). The potential pathways of extracellular superoxide production by T-G1 was tested by adding nicotinamide adenine dinucleotide phosphate (NADPH/NADP^+^), nicotinamide adenine dinucleotide (NADH/NAD^+^), and diphenyleneiodonium (DPI) chloride (Sigma, USA) to phosphate-buffered carrier solutions. Then, NADH, NADPH, and NAD^+^ were added to cell-free filtrates at a final concentration of 200 μM, and DPI was added at a final concentration of 50 μM.

### Multi-Copper Oxidase Activity Assay

Whole cell protein of T-G1 extracted from different growth phase and pH was subjected to the MCO activity assay. ABTS, [2,2′-azinobis-(3-ethylbenzthiazoline-6-sulphoate), Sigma, Germany] was used as substrate to determine the activity of laccase-like MCO activity during cell growth at pH 5.5 and 7.2. The assays were performed at room temperature with 0.5 mM ABTS in Mcilvaine buffer (mixture of 0.1 M citric acid and 0.2 M K_2_HPO_4_) using a Hach DR 3200 UV-VIS spectrophotometer (Hach Lange GmbH, Germany). Assays were initiated by adding 3 ml of enzyme solution containing 2.5 ml of Mcilvaine buffer (50 mM), 300 μL of substrate (15 mM) and 200 μL of whole cell protein as prepared above. Oxidation of the substrate was detected by determining the absorbance at 420 nm (Johannes and Majcherczyk, 2000). Data were normalized to total protein obtained from 1 OD ml^-1^ of cells (OD_600__nm_) by equation,

$$Abs=abs_{t}/V\times OD$$

Abs_t_ is the tested Abs data, V is the volume of culture from which the whole cell protein was obtained, and OD is the optical density of the culture for harvesting whole cell protein. All assays were performed in triplicate.

### MCO Gene Primer Design and qRT-PCR

The genome of *M. australicum* WSM2073^T^, the model strain of this species, is available from the GenBank database, has a size of 6,200,534 bp, and contains 6,013 protein-coding genes (genome sequence ID, NC_019973.1; Reeve et al., 2013). MCO genes were identified by key word searches against the annotated genome in the GenBank and Kyoto Encyclopedia of Genes and Genomes (KEGG) databases (Kanehisa et al., 2012). Only one laccase-like MCO gene, *mesau_02205*, was detected. The *mesau_02205* gene sequence was used to design primers Mesau_02205_163F (5′-TTCGACCTCAACCGCTACAC-3′) and Mesau_02205_163R (5′-AAACGATTGCCGAAACCTGC-3′) using DNAMAN version 8 (Lynnon Corporation). This primer set amplified a 163 bp segment of the target gene and primer specificity was checked using NCBI Primer-BLAST (Ye et al., 2012).

Polymerase chain reaction amplification was carried out in 50 μl reactions containing 25 μl Dream Taq PCR Master Mix (2X) (Thermo Scientific, Germany), 1 μl genomic DNA of *M. australicum* T-G1, and 1 nM of each primer. Thermocycling was performed with the following temperature program, an initial denaturation at 95°C for 5 min; followed by 35 cycles of denaturation at 95°C for 1 min, gradient annealing at 53, 54.4, 55.9, 57.3, 58.7, 60.1, 61.6, and 63°C for 1 min, and extension at 72°C for 1 min; and with a final extension at 72°C for 5 min. All amplifications were performed in a peqSTAR 96 × Universal Thermocycler (peQlab, UK). PCR products were analyzed by electrophoresis on a 2% agarose gel in 1 × Tris-borate-EDTA buffer stained with ethidium bromide and visualized under UV light. DNA band was cut and retrieved by GeneJET Gel Extraction and DNA Cleanup Micro Kit (Thermo, Lithuania) according to the manufacturers’ instructions. Purified PCR product was sent to Macrogen Inc. (The Netherlands) for sequencing. The sequences was compared to the GenBank database available from NCBI (Altschul et al., 1990) using the BLAST algorithm to verify specificity.

To quantify MCO gene expression, RNA was extracted from 1 ml of liquid cell culture using the TRIzol^®^ Plus RNA Purification Kit (Ambion, USA). The optical density of the culture was measured by a Hach DR 3800 UV-VIS bench top spectrophotometer (Hach Lange GmbH, Germany) before RNA extraction. Residual DNA was removed from RNA extracts with TURBO DNA-free kit (Ambion, USA). RNA extracts were used for reverse transcription to complementary DNA (cDNA) using the ArrayScript Reverse Transcriptase kit (Ambion, USA). To construct standards for quantitative PCR, 10 ng of cDNA, quantified using a Nanodrop spectrophotometer (peQlab, Germany), was used in a standard PCR using Dream Taq PCR Master Mix system (Thermo Scientific, Germany) followed by plasmid construction and analysis. Briefly, the 163 bp *mesau_02205* amplicon from T-G1 was purified with gel extraction using an agarose gel extraction kit (Jena Bioscience, Jena, Germany) and cloned using pGEM-T Easy cloning kit (Promega, USA) according to the manufacturers’ instructions. Plasmids containing the partial target gene were diluted to 10^8^ to 10^0^ copies μl^-1^ then used to construct standard curves by plotting the cycle threshold (C_T_) values vs. the standard copy number. Quantitative reverse transcription polymerase chain reaction (qRT-PCR) was performed as described before (Lu et al., 2010; Sitte et al., 2010). The annealing temperature was 57°C. Data were obtained at 72, 78, and 80°C. The C_T_ was determined automatically by the instrument. All samples were analyzed in triplicate.

### Hydrogen Peroxide and Catalase Quantification

To quantify extracellular H_2_O_2_ and catalase, T-G1 was cultured in liquid PYG media with 100 μM MnSO_4_ at pH 5.5 and pH 7.2. Spent media were sampled at exponential and stationary phases, after centrifugation at 1000 ×*g* for 5 min. The quantity of H_2_O_2_ in the supernatant of spent media was measured using the Hydrogen Peroxide Colorimetric/Fluorometric Assay Kit (BioVision, USA) according to the manufacturer’s protocol. The absorbance at 570 nm was measured with a Synergy H4 Hybrid Reader (BioTek). Catalase activity in the supernatant of spent media was measured at exponential phase and stationary phase with 10 μM H_2_O_2_ by Amplex^®^ Red Catalase Assay Kit (Life Technology) according to the manufacturer’s protocol and measuring absorbance at 560 nm. All samples were analyzed in triplicate.

### Statistical Analysis

Statistical analysis was performed with the Student’s *t*-test, one-way ANOVA, and Pearson correlation using Graphpad Prism Version 6.03. *P* values less than 0.05 were considered statistically significant. All values are averages of at least three replicates.

### Nucleotide Sequence Accession Numbers

The 16S rRNA gene sequence of *M. australicum* T-G1 was deposited in the EMBL database under the accession number HG932494. The partial sequence of the orthologs of mesau_02205 for RT-PCR was also deposited in the EMBL database under the accession number LN864498.

## Results

### Isolation and Identification of the Mn(II)-Oxidizing Bacterium T-G1 at Acidic pH

**FIGURE 1:**
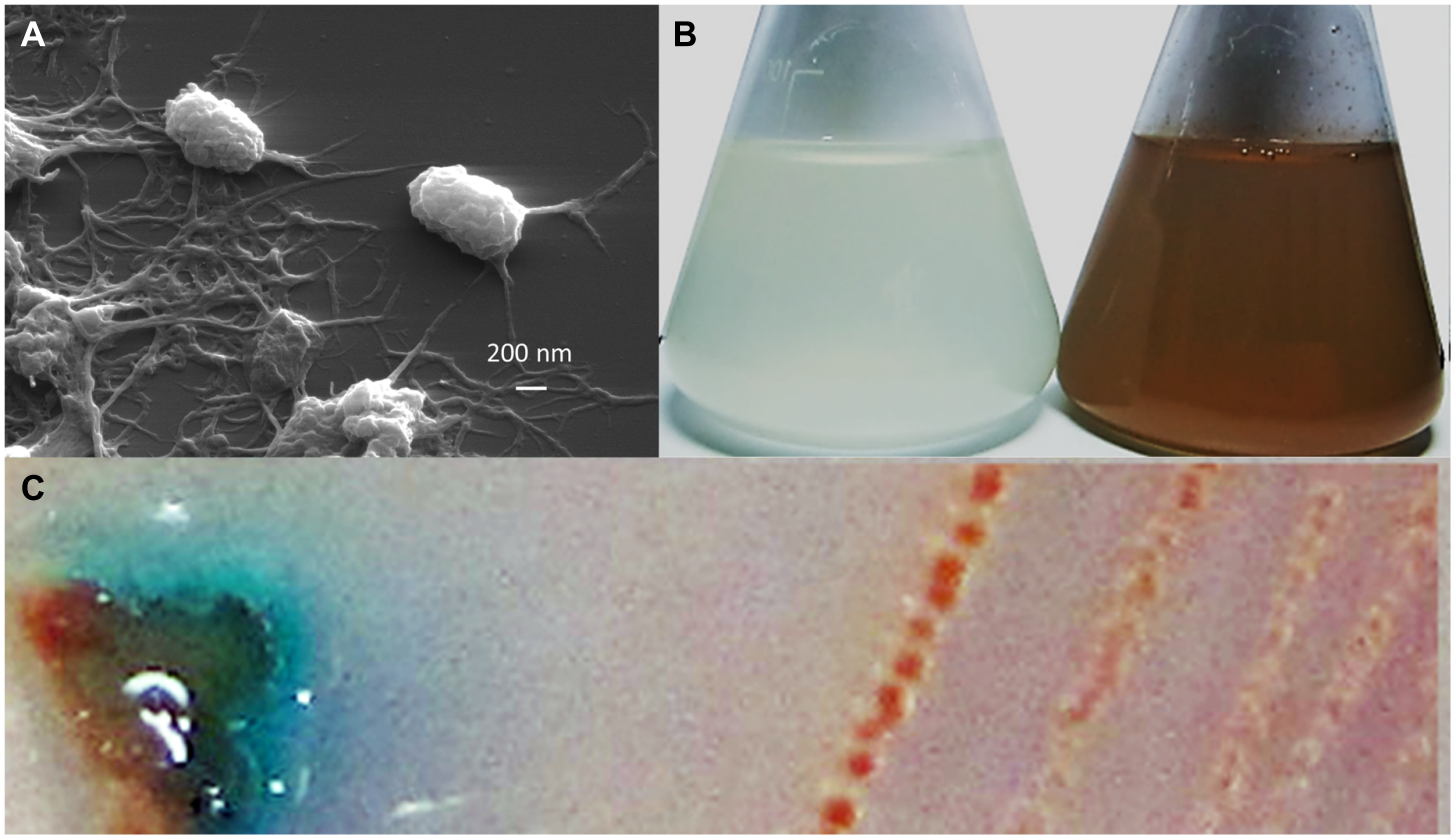
**Morphology and Mn(II) oxidation by strain T-G1: **(A)** scanning electron micrograph of T-G1 cells, showing abundant extracellular polymeric substances; **(B)** Mn(II) oxidation by T-G1 as seen by a color change in PYG medium with 10 mM MnSO_4_ (right) and without Mn (left); and **(C)** leucoberbelin blue (LBB) spot test on colony of T-G1 showing a blue color change indicative of Mn oxide production**.

**FIGURE 2:**
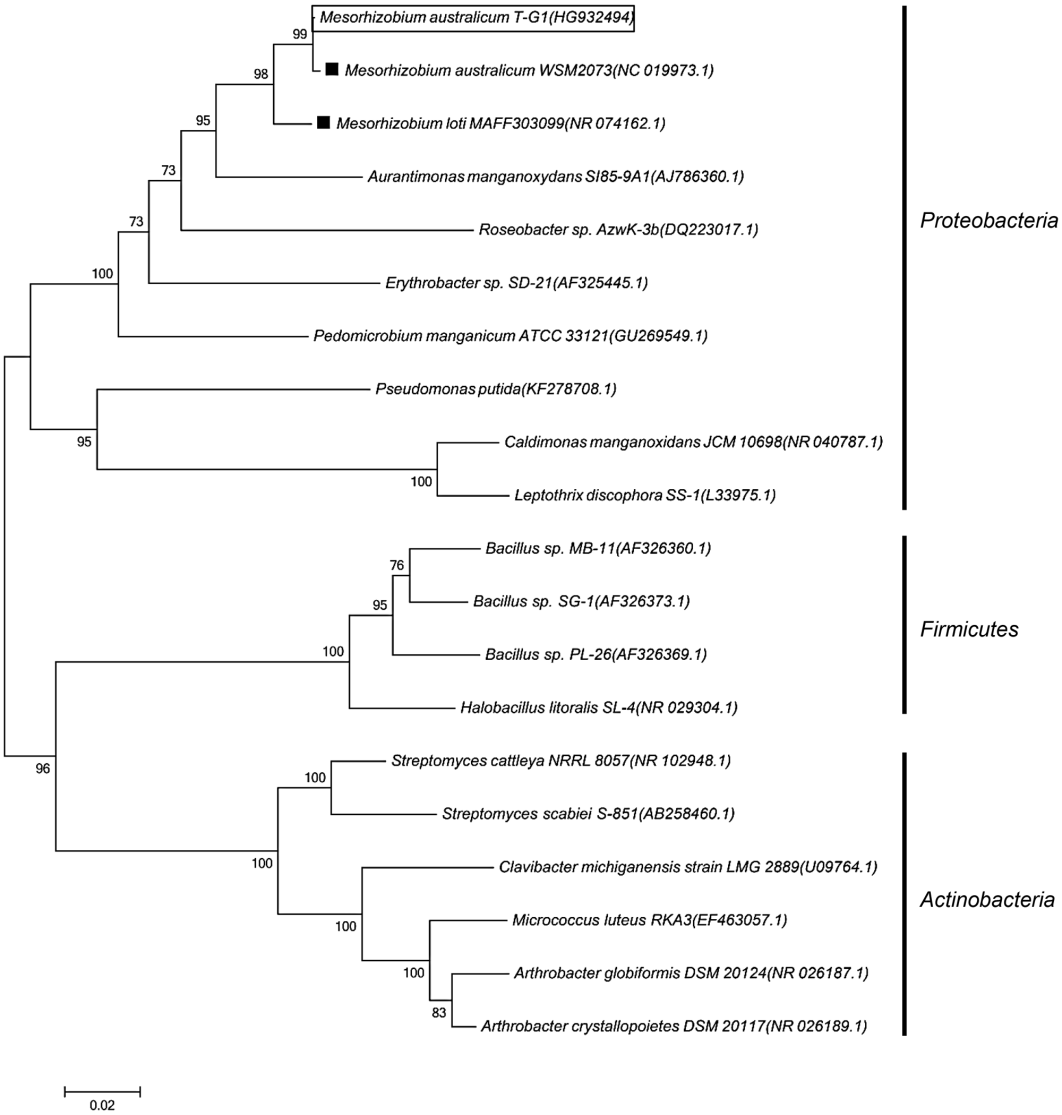
**Phylogenetic tree showing the evolutionary placement of *Mesorhizobium australicum* T-G1 (in rectangle) relative to known MOB based on 16S rRNA gene sequences**. black squares indicate non-Mn(II)-oxidizing strains. The tree was built using a neighbor joining algorithm with bootstrap values, displayed as percentages, based on 1000 replicates. Accession numbers for published sequences are shown in parentheses. The scale bar represents 0.02 substitutions per nucleotide position.

Bacterial strain T-G1 was isolated from a Mn-rich soil layer at the former uranium mining area Ronneburg, Germany. Colonies of T-G1 were round and opaque on solid PYG (pH 5.5) media. When grown in medium supplemented with Mn(II), the initial color of the colony was white, but turned orange after 7 days incubation. When grown on PYG plates without Mn(II) the colonies were pale pink after 7 days incubation. The cells were small rods (length, 0.6–1 μm; width, 200–300 nm) and surrounded by voluminous extracellular polymeric substances (**Figure 1A**). Little pH variation was observed around the colonies; pH change was from 5.5 to 5.6 ± 0.03. When cultured in liquid PYG media (pH 5.5) supplemented with a series of MnSO_4_ concentrations (0.1, 1, and 10 mM), the T-G1 culture all turned from milky to dark brown in color. No color change was observed during growth in medium without Mn(II) (**Figure 1B**). The LBB spot test for T-G1 cultured for 2 weeks on pH 5.5 PYG plates turned a dark blue color (**Figure 1C**), confirming Mn(II) oxidation at acidic pH. After 90 days incubation, no pH change was observed in liquid media. In contrast to solid-grown cultures, Mn(II) oxidation in liquid culture at pH 5.5 was not apparent until several months growth. Phylogenetic analysis of 16S rRNA gene sequences revealed that bacterial isolate T-G1 is within the Alphaproteobacteria class of the order Rhizobiales (**Figure 2**), which includes two models MOB, Aurantimonas manganoxydans SI85-9A1 and Roseobacter sp. AzwK-3b. Further, the isolate clustered with the genus Mesorhizobium and was most closely related to the mesophile, *M. australicum* WSM2073 (99% 16S rRNA gene sequence similarity).

### *M. australicum* Strain T-G1 Mediated Mn Oxide Formation at pH 5.5

**FIGURE 3:**
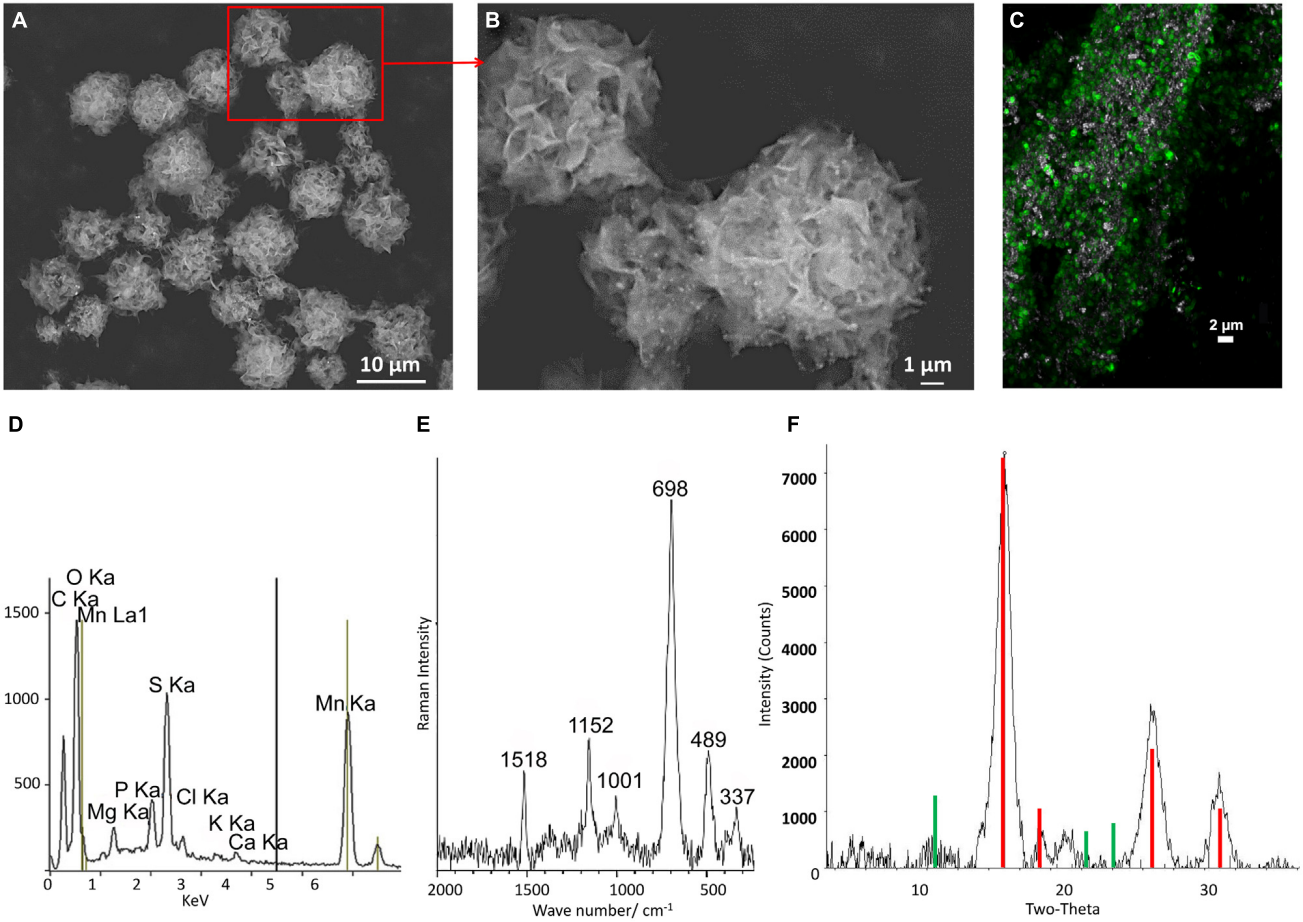
**Characterization of biogenic Mn oxides produced by strain T-G1 in PYG medium at pH 5.5. (A)** Scanning electron micrograph showing spherical clusters of micron to sub-micron sheets of Mn oxide minerals. **(B)** An enlargement of the area indicated by a red box in **(A)**. **(C)** Confocal laser scanning microscopy (CLSM) of a mineral aggregate in association with T-G1. Bacterial cells stained by FM 1-43 probe shown in green, reflection of minerals was indicated in gray. **(D)** Energy-dispersive X-ray (EDS) spectra showing elemental composition of biogenic Mn oxide particles. **(E)** Raman spectroscopy of biogenic Mn oxides. **(F)** XRD pattern of biogenic Mn-oxide minerals with vertical lines indicating strong (red) and weak peak positions (green) of bixbyite.

Biogenic Mn oxide minerals produced by T-G1 occurred as discrete, micron-scale spheres of thin, wispy sheets of crystals (**Figures 3A,B**). There was additional nanometer scale particles associated with these larger spheres that were too small to be analyzed further. T-G1 cells were homogeneously distributed and colonized the surface of Mn oxides as shown by CLSM (**Figure 3C**). Qualitative chemical composition, detected by EDS, showed that the phase was composed mainly of Mn, O, C, S (most likely from the MES buffer) and P, with minor amounts of Mg, K, and Ca (**Figure 3D**). The Raman spectra were dominated by six intense bands at 337, 489, 698, 1001, 1152, and 1518 cm^-1^ (**Figure 3E**). While the last three Raman bands can be assigned to carotenoids present in the bacterial cells (Ciobotã et al., 2012), the assignment of the other three bands, located at wavenumbers smaller than 1000 cm^-1^, is much more challenging. The Raman bands at 337, 489, and 698 cm^-1^ are tentatively assigned to a bixbyite-type mineral. The differences in the position and intensity of the Raman bands of the T-G1 biogenic minerals and those previously reported for bixbyite (analogic peak positions, 314, 481, and 698 cm^-1^; Julien et al., 2004) could be explained by the influence of the other elements incorporated in the crystal structure of the bixbyite-type mineral, as revealed by EDS investigations. XRD patterns of the biogenic Mn oxide minerals had intense peaks at angles (2θ = 15°, 25°, **Figure 3F**), which confirmed the formation of bixbyite-like Mn oxide (Baldi et al., 1998). The peaks, especially at 7 and 10 Å, indicating a layered birnessite-like structure were absent.

### Linking Mn(II) Oxidation to ROS and MCO Expression at Different pH and Growth Phases

**FIGURE 4:**
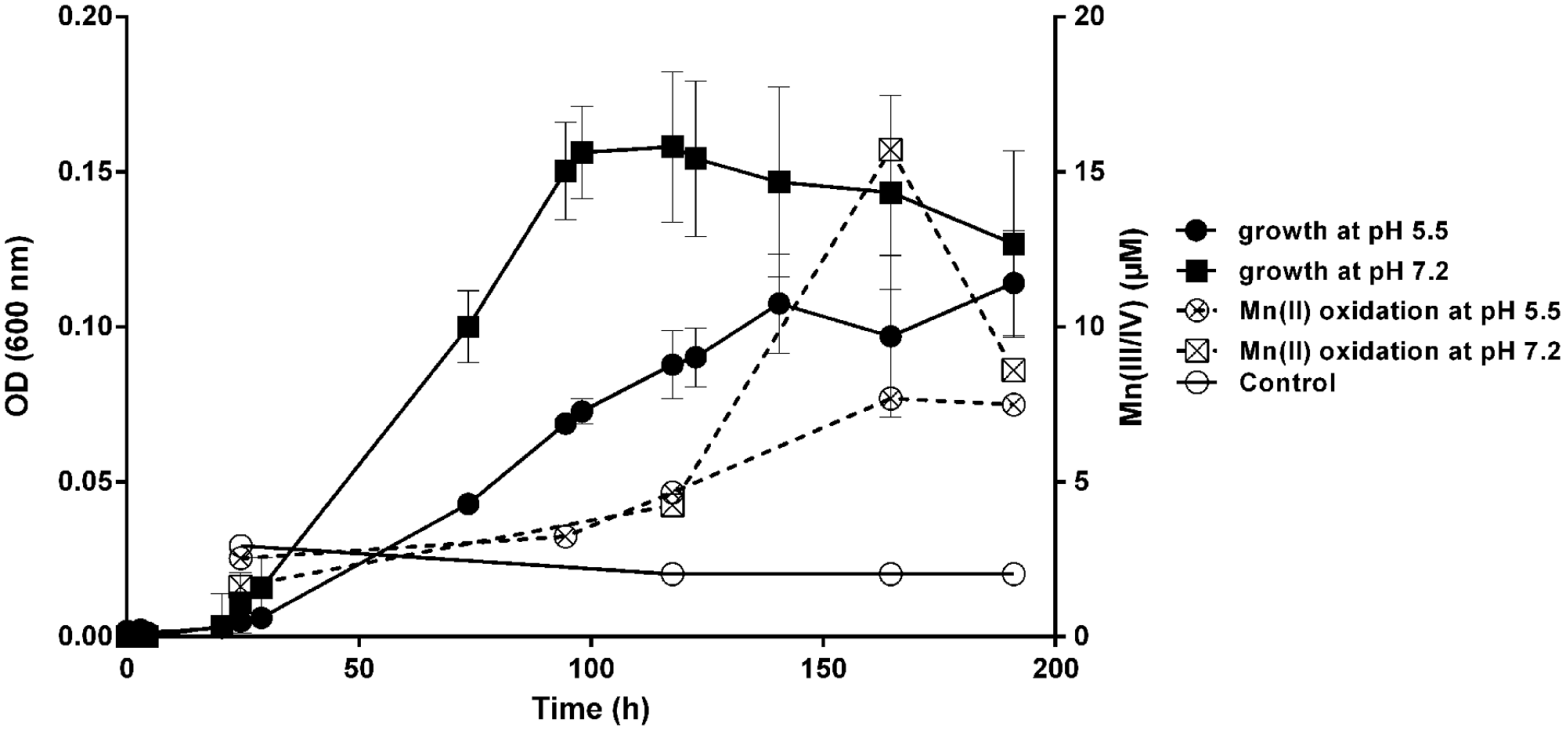
**Mn(II) oxidation and growth of T-G1 at different pH values.** Growth (OD_600_) and Mn(II) oxidation at pH 5.5 and pH 7.2. The data for the Mn(II) oxidation control at pH 7.2 overlapped with that at pH 5.5, therefore, only the data at pH 5.5 is shown. Error bars represent SD.

The ability of *M. australicum* T-G1 to oxidize Mn(II) was dependent on the medium pH and growth phases (**Figure 4**). The rate of Mn(II) oxidation at pH 7.2 increased sigmoidally, beginning from the early stationary phase. In contrast, at pH 5.5, Mn(II) oxidation occurred with a slow linear rate from early exponential phase to late stationary phase and achieved a maximum rate at early stationary phase. Average Mn(II) oxidation rates at pH 5.5 and pH 7.2 were 0.032 ± 0.0087 μM h^-1^ and 0.11 ± 0.028 μM h^-1^ (average rate from 24.5 to 164.5 h), respectively. Intriguingly, in late stationary phase (around 191 h incubation), the concentration of Mn(III/IV) in the pH 7.2 treatment decreased dramatically to 54.74% compared to that in 164.5 h incubation. In contrast, Mn(III/IV) concentrations only decreased 2.59% after 191 h incubation at pH 5.5. Pearson correlation coefficient of Mn(II) oxidation and cell growth at pH 5.5 was 0.897 whereas it was 0.58 at pH 7.2. This indicates that Mn(II) oxidation by T-G1 at acidic pH was closely associated with cell growth, but at neutral pH there was no strong linear correlation.

**FIGURE 5:**
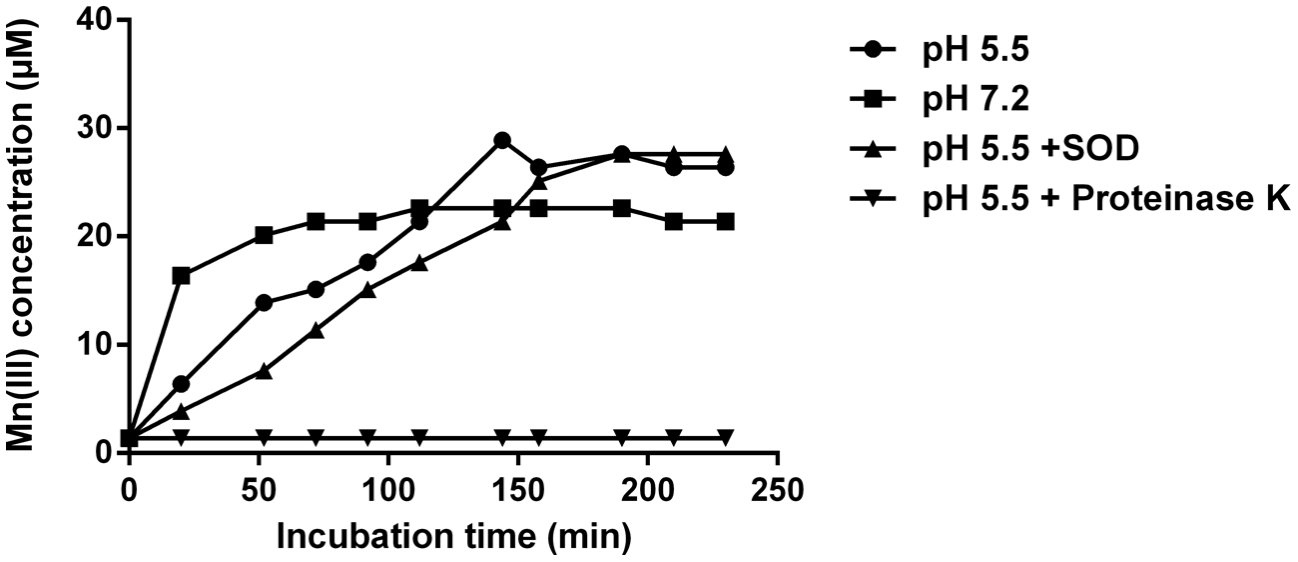
**Mn(III) trapping by 100 μM pyrophosphate during incubation of cell-free filtrate with 100 μM MnSO_4_ at pH 5.5, pH 7.2 and with the addition of superoxide dismutase (SOD, 5 μM) or proteinase-K (100 μg ml^-1^).** Error bars represent SD and are smaller than symbols.

Mn(III) pyrophosphate trapping experiments showed that Mn(III) production occurred at rates of ∼0.2 μM min^-1^ and 0.4 μM min^-1^ at acidic and neutral pH, respectively (**Figure 5**). At pH 5.5 in the presence of SOD, the rate of Mn(III) formation was lower (0.14 μM min^-1^). However, an unpaired *t*-test showed no significant difference between the treatments with and without SOD at pH 5.5 (P = 0.5823). Enzymatic activity was required for the production of Mn(III), as Mn(II) oxidation was inhibited and no measurable Mn(III) was produced in the presence of proteinase K (**Figure 5**).

**FIGURE 6:**
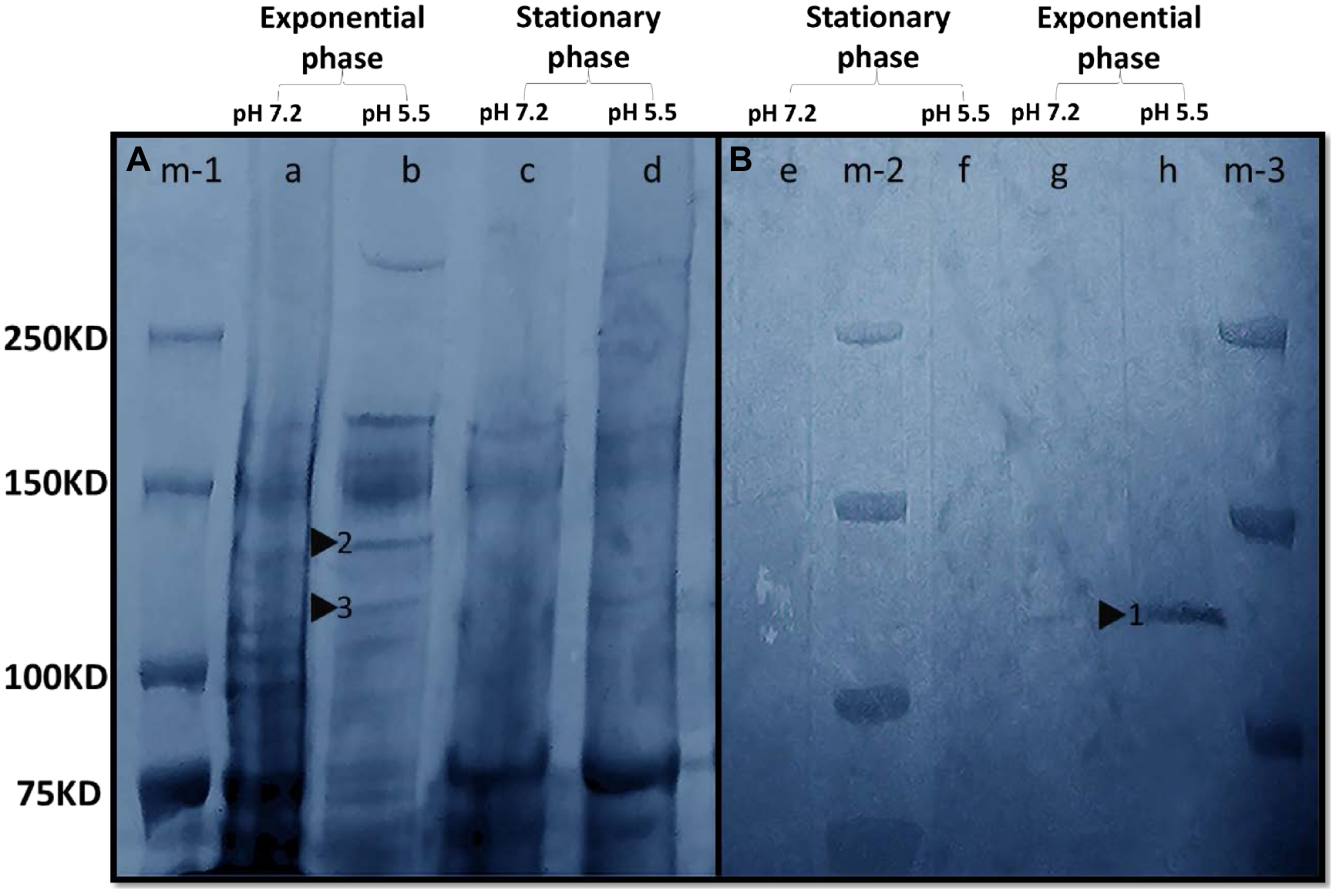
**In-gel activity of Mn(II) oxidation enzymes. (A)** Gel was stained with Coomassie. Lane m-1, Precision Plus Protein Dual Xtra Standards; Lane a, whole cell protein (pH 7.2, exponential phase); Lane b, whole cell protein (pH 5.5, exponential phase); Lane c, whole cell protein (pH 7.2, stationary phase); Lane d, whole cell protein (pH 5.5, stationary phase). Triangles 2 and 3 indicated the putative Mn(II) oxidizing enzymes with molecular masses of 140 and 120 KDa, respectively. **(B)** Gel was stained with 200 μM MnSO_4_. The color of the gel was modified to blue in Adobe Photoshop CC to enhance the clarity of the bands. Lane m-2 and m-3, Precision Plus Protein Dual Xtra Standards; Lane e, whole cell protein (pH 7.2, stationary phase); Lane f, whole cell protein (pH 5.5, stationary phase); Lane g, whole cell protein (pH 7.2, exponential phase); Lane h, whole cell protein (pH 5.5, exponential phase). Triangle 1 indicated the putative Mn(II) oxidizing enzyme with a molecular mass between 120 and 140 KDa.

Sodium dodecyl sulfate polyacrylamide gel electrophoresis revealed that *M. australicum* T-G1 had different protein expression patterns depending on the pH and growth phase (**Figure 6**). T-G1 had a more active metabolism in exponential phase with a variety of protein bands detected both at pH 5.5 and pH 7.2. Two bands between 120 to 140 KD were detected in the exponential phase at pH 5.5. During the stationary phase at pH 5.5, a weak band (lane d, **Figure 6A**) was observed around 140 KD. A MnSO_4_ staining positive band was detected within the area of the gel (**Figure 6B**, signal indicated by black triangle) with a similar size to the full length MCO MnxG protein (138 KD, a putative Mn oxidizing enzyme from marine spore-forming Bacillus sp. SG-1; van Waasbergen et al., 1996). Surprisingly, the positive signal of MnSO_4_ staining was not present at pH 7.2 or in the stationary phase at pH 5.5, suggesting that growth phases and pH may regulate Mn(II) oxidase expression. In addition, PAGE experiments revealed that NBT positive bands could be clearly detected in the stationary phase at pH 7.2 but not at pH 5.5 indicating the presence of superoxide under neutral pH conditions (data not shown).

**FIGURE 7:**
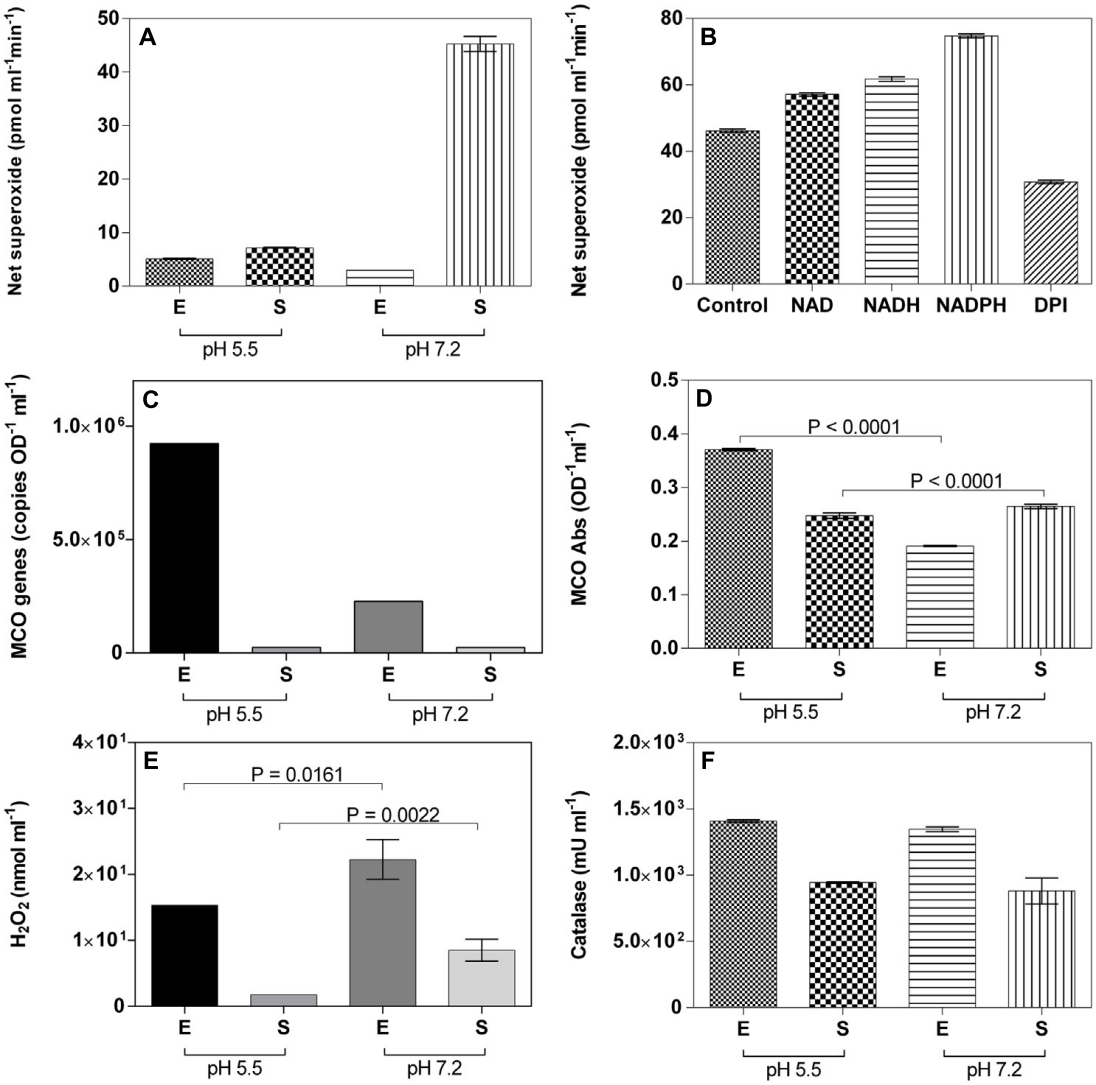
**Reactive oxygen species, MCO, H_2_O_2_, and catalase quantification during exponential (E) or stationary (S) growth at pH 5.5 or 7.2. (A)** Superoxide quantification during different growth phases; **(B)** Superoxide quantification in the presence of NAD, NADH, NADPH, and DPI at pH 7.2; **(C)** Quantification of MCO gene copies using qRT-PCR; **(D)** MCO activity; **(E)** H_2_O_2_ quantification during different growth phases; **(F)** Catalase quantification during different growth phases. Error bars represent SD.

Superoxide measurements during growth of *M. australicum* T-G1 showed that the strain consistently produced the highest amount of ROS in early stationary phase at pH 7.2 (**Figure 7A**), similar to the model MOB Pseudomonas putida GB-1 (Diaz et al., 2013). Meanwhile, *M. australicum* T-G1 produced superoxide at pH 5.5 during both exponential and early stationary phase, but the amount was much lower relative to the stationary phase at pH 7.2; interestingly, the lowest amount of superoxide was produced during exponential phase at pH 7.2. Considering that the highest production of net superoxide occurred in early stationary phase, there seemed to be a sudden production of superoxide by *M. australicum* T-G1 during growth at neutral pH. Moreover, the production of superoxide at pH 7.2 corresponded with increased Mn(III/IV) concentration in early stationary phase. Addition of the reduced cofactor NADH and NADPH and oxidized cofactor NAD^+^ substantially increased superoxide production [F(4,10), p < 0.0001; **Figure 7B**]. Addition of DPI, a known inhibitor of NAD(P)H oxidoreductase and other NAD(P)H oxidizing enzymes, notably decreased superoxide production (**Figure 7B**).

Only one laccase-like MCO gene, mesau_02205, was detected in the draft genome of *M. australicum* WSM2073^T^, a phylogenetically related organism to T-G1. Using the qRT-PCR approach developed in our study, we showed that *M. australicum* T-G1 vigorously expressed the MCO gene during the exponential phase at low pH. Expression of the gene at pH 5.5 (9.24 × 10^5^ OD^-1^ ml^-1^) was four times higher than that at pH 7.2. However, on average only 2.47 × 10^4^ OD^-1^ ml^-1^ of mesau_02205 was obtained in stationary phase both at pH 5.5 and pH 7.2 (**Figure 7C**). Consistent with the results of qRT-PCR, the highest MCO activity was detected in exponential phase at pH 5.5 (**Figure 7D**). MCO obtained from exponential phase at pH 5.5 catalyzed twofold higher ABTS than the MCO from pH 7.2. Additionally, in exponential phase, an unpaired *t*-test revealed that the difference in MCO production between pH 5.5 and pH 7.2 was significant (P < 0.0001). This result was in line with the qRT-PCR assay. In contrast, the activity of MCO both at pH 5.5 and pH 7.2 in stationary phase was relative strong compared to the low expression of the MCO gene. The difference in activity between pH 5.5 and pH 7.2 was significant as well (P < 0.0001; **Figure 7D**).

Quantification of extracellular H_2_O_2_ revealed a higher amount of H_2_O_2_ in the spent media of T-G1 at pH 7.2 compared to spent media at pH 5.5 in the exponential phase (**Figure 7E**). An unpaired *t*-test showed that this difference was significant (*P* = 0.0161). At stationary phase, the amount of H_2_O_2_ produced at neutral pH was statistically different than at acidic pH (*P* = 0.0022). Consistent with the concentrations of H_2_O_2_ in the exponential phase, the extracellular activity of catalase in the spent media at pH 5.5 was 1407 ± 10 mU ml^-1^, slightly higher than at pH 7.2 which was 1347 ± 17.3 mU ml^-1^ (**Figure 7F**). However, in the stationary phase, the activity of catalase was not significantly different between pH 5.5 and pH 7.2.

## Discussion

Biological Mn oxidation is an important pathway of the global Mn cycle and can also impact carbon cycling by increasing substrates for Mn-reducing bacteria (Nealson and Myers, 1992) and because Mn oxide minerals catalyze the degradation of refractory compounds (Tebo et al., 2004). In addition, biological Mn oxidation can contribute to the remediation of heavy metal contaminants (Burkhardt et al., 2009; Luan et al., 2012; Chaput et al., 2015). Knowledge of the microbial Mn oxidation process is limited to circumneutral pH environments where biological Mn oxidation is thermodynamically favorable when the two electrons transfer steps are coupled. In acidic environments where reactive oxygen species are unstable, Mn(III/IV) oxide formation is thermodynamically unfavorable when taking into account O_2_ as the major terminal electron acceptor (Luther, 2005, 2010) and there are few bacterial isolates available for investigating the pathways of low pH Mn(II) oxidation. In this study, we isolated a new low pH MOB strain, *M. australicum* strain T-G1, from acidic, Mn-rich subsoils at a former uranium leaching heap. This organism grows at both acidic and neutral pH and provided the first glimpse into microbial mechanisms for initiating Mn oxide formation at both acidic and neutral pH.

### The Binary Mn(II) Oxidation Mechanism

For T-G1 growing at low pH, Mn oxidation likely occurs through the MCO pathway, whereas, enzymatically produced ROS might be responsible for a higher rate of Mn oxide formation during early stationary phase growth under neutral pH condition. This differs from previous reports where MCOs were demonstrated to participate in Mn(II) oxidation by model MOB, such as *Bacillus* sp. strain SG-1 (Francis et al., 2002), *Leptothrix discophora* SS-1 (Corstjens et al., 1997), and *Pedomicrobium* sp. strain ACM 3067 (Anderson et al., 2009) at neutral pH. Recently, Geszvain et al. (2013) also reported a two-component gene cluster in *P. putida* GB-1 regulates Mn(II) oxidation, which was composed by PputGB1_2447 and PputGB1_2665. These genes encode two MCO enzymes that each independently are capable of oxidizing both Mn(II) and Mn(III) (Geszvain et al., 2013). However, alternative enzymatic pathways promoting Mn(II) oxidation are observed by different *Alphaproteobacteria* species. For example, *A. manganoxydans* SI85-9A1, is catalyzed by *mopA*, a heme peroxidase-like enzyme (Anderson et al., 2009), while *Roseobacter* sp. AzwK-3b oxidizes Mn(II) using a superoxide pathway (Learman et al., 2011) and potentially in coordination with a heme peroxidase although the specific pathways are still under investigation.

*Mesorhizobium australicum* T-G1 can initiate Mn(II) oxidation by varying its oxidation mechanisms depending on the pH. The binary Mn(II) oxidization pathways used by T-G1 could include survival mechanisms for life in the oligotrophic and acidic subsoils at the former uranium leaching heap, which experience fluctuating groundwater levels (Grawunder et al., 2009). Groundwater fluctuations at this site likely leads to variability in environmental parameters, and T-G1 must be poised to shift Mn(II) oxidation mechanisms with shifting conditions such as pH changes. We observed that the expression of the laccase-like MCO aligned with Mn(II) oxidation at pH 5.5 rather than at pH 7.2. Similarly, Kim et al. (2001) elucidated that *clac2*, an acidic laccase (a type of MCO) gene from *Coprinus congregatus* had four times higher expression at pH 4.1 than neutral pH (Kim et al., 2001). Previous observations also revealed that majority of known laccases are more stable at acidic pH (He et al., 2014).

Though the MCO pathway appears to be a dominant process for T-G1 to catalyze low pH Mn(II) oxidation, the strain also plausibly oxidized Mn(II) at a higher rate, through extracellular superoxide radicals. Superoxide radicals were produced in abundance during the beginning of stationary phase at neutral pH, then followed a sharp decrease in the amount of Mn(III/IV) at late stationary phase. As is the case for T-G1, *Escherichia coli* cells are subject to a sudden burst of oxidative stress during the early stationary phase that affects a large number of proteins. It was believed to be triggered by programmed cell death or the formation of “viable but nonculturable” (VBNC) cells (Thomas, 2005). However, the stimulation by NADH and NADPH and inhibition by DPI suggests that the biochemical process responsible for superoxide production in T-G1 might also be a nonspecific response as seen with other ROS producing bacteria (Diaz et al., 2013). Constrained experiments with selected stress conditions need to be performed with T-G1 to further elucidate the impact of abiotic and biotic factors on superoxides production.

### Low Rate of Mn(II) Oxidation at Acidic pH

Generally, external pH can affect the metabolic properties of bacterial cells (Booth, 1985). For instance, enzyme expression for carbon metabolism in *E. coli* responds differently to low and high medium pH (Gale and Epps, 1942; Castellanos-Mendoza et al., 2014). Interestingly, we observed that T-G1 had the same growth rate at pH 5.5 and pH 6.8 when 500 μM MnSO_4_ was added to the media. However, in contrast to Mn(II) oxidation at neutral pH, T-G1 initiated the Mn oxidation reaction at pH 5.5 at a comparatively low rate (0.032 ± 0.0087 μM h^-1^ average rate from 24.5 to 164.5 h) relative to oxidation at neutral pH. Previous work revealed that O_2_ can abiotically oxidize Mn(H_2_O)_6_^2+^ merely after the hydration water is replaced by inorganic (e.g., OH^-^, increase pH) or organic ligands (Luther, 2005). However, the first step in oxidizing Mn(II) to Mn(III) with O_2_ is rate limiting (Luther, 2010). In acidic environments, the high amount of H^+^ or H_3_O^+^ may associate with Mn(H_2_O)_6_^2+^, making the replacement of hydration water to OH^-^ more difficult, further limiting the reaction rate (Rossëinsky, 1963). Consequently, Mn oxide formation in liquid culture at low pH is slow and it often took months to be visible.

### Potential Benefits for T-G1 to Oxidize Mn(II)

T-G1 is a strain of *M. australicum*, a known N_2_-fixing bacterium that has not been shown to oxidize Mn previously (Nandasena et al., 2009) although its genome harbors a laccase-like MCO gene (*mesau_02205*, Gene ID 14399755). However, a N_2_-fixing species, *Rhizobium* sp. M4, isolated from the Mary Kathleen uranium mine, which is rich in Mn, is within the same Order (*Rhizobiales*) as T-G1 and was identified as a MOB (Moy et al., 2003). Though there is no clear evidence linking biological Mn(II) oxidation to N_2_ fixation process, there are still several potential benefits for N_2_-fixing bacteria to oxidize Mn(II). First, N_2_ might be released from the degradation of refractory organics with Mn oxides in nitrogen limiting deep subsurface niches. Second, the reduced manganese formed in the above reaction could readily react with ROS to protect nitrogenase from oxidative toxicity (Fay, 1992; Daly, 2009). In addition, these two reactions together are more thermodynamic favorable than the oxidation of organic matter by O_2_ which indicate potential energy conservation through this process in microaerobic environments (Luther et al., 1997).

### The Ecophysiological Importance of Mn(III) Oxides

At low pH, MCO might be the dominant pathway for T-G1 to initiate the bixbyite-like Mn(III) mineral formation. The oxidation of Mn(II) to Mn(IV) proceeds with two consecutive one electron transfers with Mn(III) as an intermediate. Under neutral to alkaline condition, Mn(III) can rapidly disproportionate to Mn(IV), leaving little time for Mn(III) to accumulate to detectable amounts. It is observed, however, that Mn(III) is stabilized at low pH (4-5) or when associated with certain chelating compounds like polysaccharides (Davies, 1969). Webb et al. (2005) verified that Mn(III) can be stabilized in solution when linked to ligands such as pyrophosphate. Consistently, soluble Mn(III) complexes have been detected as the dominant dissolved Mn species in suboxic water columns of oceanic and sediment habitats (Madison et al., 2013). Furthermore, the reaction of Mn(II) with superoxide could maintain a significant fraction of dissolved Mn in the +III oxidation state (Hansard et al., 2011). Mn(III) trapping experiments of T-G1 revealed that enzymatic generation of Mn(III) could also be detected at acidic pH. However, superoxide had no major effect on Mn(III) formation at pH 5.5 indicating other factors such as MCO may lead this process at low pH. Interestingly, Mn(III) is the primary Mn oxidation state found in the bixbyite-like Mn oxide produced by T-G1. Therefore, Mn(III) might be stabilized and immobilized as a solid phase in this biogenic mineral.

Mn(III) minerals can be formed through both abiotic and biotic pathways (Mandernack et al., 1995; Bargar et al., 2005; Spiro et al., 2010; Hosseinkhani and Emtiazi, 2011; Lefkowitz et al., 2013; Furuta et al., 2014; Zhang et al., 2014). Bixbyite-like Mn_2_O_3_ minerals can easily form through oxidative precipitation when high-valence manganese species, e.g., Mn(VII), are applied at low pH (Freitas et al., 2013). *Bacillus* sp. (Zhang et al., 2014) and *Acinetobacter* sp. (Hosseinkhani and Emtiazi, 2011) can initiate formation of bixbyite-like Mn_2_O_3_ minerals at pH 7.5. *Bosea* sp. strain BIWAKO-01 is the only known bacterium which can mediate bixbyite formation at slightly acidic pH 6.0–6.3 (Furuta et al., 2014). Although Lefkowitz et al. (2013) noted that there is no transformation from birnessite [predominantly Mn(IV)] to Mn(III) minerals at pH < 7, whether or not the bixbyite-like mineral is a product of a secondary reaction at acidic pH is still uncertain. Nevertheless, birnessite and todorokite rather than bixbyite were assumed to be the main Mn oxides in the Mn-rich layer at the former uranium leaching heap (Schäffner, 2013).

Surprisingly, we did not observe formation of Mn(III/IV) oxide particles in T-G1 liquid cultures at pH 7.2 despite the positive LBB test and Mn(III) trapping results. According to the Learman et al. (2013) study with Mn(II)-oxidizing *Roseobacter* sp., Mn (III/IV) oxides were formed only in the presence of active catalase, which decomposes H_2_O_2_ produced when superoxide serves as a terminal electron acceptor during Mn(II) oxidation reactions (Learman et al., 2013). It is assumed that H_2_O_2_ inhibits Mn(II) oxidation by reducing Mn(III). Indeed, cultures of T-G1 had statistically significant higher amounts of H_2_O_2_ and lower activity of catalase at pH 7.2 than those at pH 5.5 which might partially explain the lack of Mn(III/IV) oxide particles at neutral pH. It is possible that either the effect of low pH or abundant polysaccharides produced by T-G1 may stabilize Mn(III) in the complex system. The presence of soluble Mn(III) has important environmental ramifications. For example, Mn(III) is the primary oxidant in many systems (Johnson and Xyla, 1991; Manceau et al., 1997; Nico and Zasoski, 2000) and is more active than Mn(IV) in redox reactions (Fredrickson et al., 2002).

## Conclusion

The ecophysiological influence of Mn oxides may be more significant than often acknowledged by their relatively low abundance comparing to other major minerals like quartz, illite, kaolinite or feldspars in terrestrial environments. Especially, in the former uranium leaching heap, Mn oxides may serve as the main oxidant and scavenger of trace nutrients to sustain the biosphere in this extreme oligotrophic environment. We show here that *M. australicum* T-G1 has pH-dependent Mn(II) oxidation strategies and initiates bixbyite-like mineral formation under acidic conditions that might be a possible origin of Mn oxides found in subsurface Mn-rich layers at the former uranium leaching heap.

## Author Contributions

Study conception and design: TB, CS, DA, KK. Administrative support: CS, DA, KK. Collection and assembly of data: TB, CS, TN, VC, PR, JP, SN. Data analysis: TB, CS, TN, VC, PR, JP, SN. Data interpretation: TB, CS, TN, VC, PR, JP, SN, DA. Manuscript drafting: TB, CS, DA, TN, VC, KK. Critical revisions to the manuscript: TB, CS, DA, TN, KK.

## Conflict of Interest Statement

The authors declare that the research was conducted in the absence of any commercial or financial relationships that could be construed as a potential conflict of interest.
