# Supplementary material for: Characterization of pH dependent Mn(II) oxidation strategies and formation of a bixbyite-like phase by Mesorhizobium australicum T-G1
Source: Front Microbiol. 2015 Jul 17;6:734. doi: 10.3389/fmicb.2015.00734 (PMC4505141; doi:10.3389/fmicb.2015.00734)
Supplement: Supplementary file 1 [file Data_Sheet_1.DOCX]

***Supplementary Material***

**Characterization of pH dependent Mn(II) oxidation strategies and formation of a bixbyite-like phase by *Mesorhizobium australicum* T-G1**

**Tsing Bohu^1^, Cara M. Santelli^2^, Denise M. Akob^3^, Thomas R. Neu^4^, Valerian Ciobota^5^, Petra Rösch^5^, Jürgen Popp^5, 6^, Sándor Nietzsche^7^, and Kirsten Küsel^1, 8*^**

^1^Aquatic Geomicrobiology, Friedrich Schiller University Jena, Jena, Germany

^2^Department of Mineral Sciences, Smithsonian Institution, Washington, DC, USA

^3^National Research Program, U.S. Geological Survey, Reston, VA, USA

^4^Department of River Ecology, Helmholtz Centre for Environmental Research-UFZ, Magdeburg, Germany

^5^Institute of Physical Chemistry and Abbe School of Photonics, Friedrich Schiller University Jena, Jena, Germany

^6^Institute of Photonic Technologies, Jena, Germany

^7^Centre of Electron Microscopy, University Hospital Jena, Friedrich Schiller University Jena, Jena, Germany

^8^German Centre for Integrative Biodiversity Research (iDiv) Halle-Jena-Leipzig, Leipzig, Germany

*** Correspondence:** Kirsten Küsel, Aquatic Geomicrobiology, Friedrich Schiller University Jena, Dornburger Strasse 159, Jena, 07743, Germany.

kirsten.kuesel@uni-jena.de

1. **Supplementary Tables**

Table S1 Physicochemical data of the soil and porewater from the Mn layer

| Sample | pH | C | N | Mn | Fe | Cd | Zn | As | Co | Cr | Cu | U |
| --- | --- | --- | --- | --- | --- | --- | --- | --- | --- | --- | --- | --- |
| Soil  [μg/g] | 4.3 | 500 | 300 | 7550 | 33945 | 1.34 | 91.6 | 10.1 | 27.8 | 39 | 40.1 | 2.7 |
| Porewater  [μg/L] | 4-5 | nd | 280-2800 | 35556 | 267 | 50 | 1667 | 10 | 1778 | 3.6 | 53.3 | 3.3 |

Note: The porewater data were retrieved from (Burkhardt et al., 2009). nd: not detected

**Reference:**

Burkhardt, E.-M., Meißner, S., Merten, D., Büchel, G., and Küsel, K. (2009) Heavy metal retention and microbial activities in geochemical barriers formed in glacial sediments subjacent to a former uranium mining leaching heap. *Chem. Erde - Geochem.* **69**: 21–34.
